# The impact of COVID-19 on the mental health of Lebanese pharmacists: A national cross-sectional study

**DOI:** 10.3389/fpubh.2023.1156840

**Published:** 2023-04-13

**Authors:** Jihan Safwan, Dalal Hammoudi Halat, Marwan Akel, Samar Younes, Mohamad Rahal, Nisreen Mourad, Zeina Akiki, Michelle Cherfane, Faraj Saade, Etwal Bouraad, Mariam Dabbous, Fouad Sakr

**Affiliations:** ^1^Biomedical Sciences Department, School of Pharmacy, Lebanese International, University, Beirut, Lebanon; ^2^INSPECT-LB (Institut National de Santé Publique, d’Épidémiologie Clinique et de Toxicologie-Liban), Beirut, Lebanon; ^3^Academic Quality Department, QU Health, Qatar University, Doha, Qatar; ^4^Pharmaceutical Sciences Department, School of Pharmacy, Lebanese International, University, Beirut, Lebanon; ^5^Pharmacy Practice Department, School of Pharmacy, Lebanese International, University, Beirut, Lebanon; ^6^School of Education, Lebanese International University, Beirut, Lebanon; ^7^Population Health Division, Gilbert and Rose-Marie Chagoury School of Medicine, Lebanese American University, Byblos, Lebanon; ^8^Alice Ramez Chagoury School of Nursing, Lebanese American University, Byblos, Lebanon; ^9^Lebanese Order of Pharmacists, Beirut, Lebanon; ^10^Department of Epidemiology and Population Health, American University of Beirut, Beirut, Lebanon; ^11^UMR U955 INSERM, Institut Mondor de Recherche Biomédicale, Université Paris-Est, Créteil, France; ^12^École Doctorale Sciences de la Vie et de la Santé, Université Paris-Est, Créteil, France

**Keywords:** COVID-19, frontline, Lebanon, pharmacists, mental health

## Abstract

**Introduction:**

The COVID-19 pandemic has induced a global mental health crisis with variable consequences. This study aimed to assess the psychological impact of COVID-19 regarding anxiety, insomnia, depression, and response to trauma on pharmacists in Lebanon during COVID-19, and to identify factors contributing to psychological distress.

**Methods:**

This was a cross-sectional study among pharmacists that involved the use of the 7-item Generalized Anxiety Disorder (GAD-7), 7-item Insomnia Severity Index (ISI), Patient Health Questionnaire 9-item depression module (PHQ-9), and Impact of Event Scale revised (IES-R) subscales. Descriptive statistical analyses were performed to determine the study distribution. The associations between the scores and the participants’ characteristics were assessed using the Chi-square test. Four binary logistic regression models were used to evaluate the association between the scores and the potential confounders, followed by four multivariable logistic regressions. An alpha of 0.05 was used to determine statistical significance.

**Results:**

Participants comprised 311 pharmacists from all Lebanese districts, of whom 251 (80.7%) were females and 181 (58.2%) aged between 26 and 35 years. The majority of the participants were community pharmacists (*n* = 178, 57.2%). A considerable proportion of participants had symptoms of anxiety (*n* = 128, 41.2%), insomnia (*n* = 64, 20.6%), depression (*n* = 157, 50.5%), and subjective stress (*n* = 227, 78.8%). Higher anxiety (aOR: 1.73, 95% CI: 1.08; 2.78, *p*-value: 0.02), higher depression (aOR: 3.06, 95% CI: 1.73; 5.39, p-value: 0.001), and higher stress (aOR: 1.86, 95 percent CI: 1.11; 3.14, p-value: 0.02) scores were significantly associated with pharmacists who reported that their work involves contact with infected/suspected COVID-19 patients. Interestingly, pharmacists who expressed concern about contracting COVID-19 infection had significantly higher anxiety (aOR: 2.35, 95% CI: 1.40; 3.94, *p*-value: 0.001) and higher depression scores (aOR: 2.64, 95% CI: 1.49; 4.67, p-value: 0.001) respectively.

**Conclusion:**

The preliminary results from pharmacists in Lebanon reflect increase in stress, burden, and frustration felt by pharmacists, creating a negative impact on their mental health and well-being during the global pandemic. As frontline healthcare workers, the role of pharmacists in the community should not be overlooked, and their mental health should be well investigated.

## Introduction

On December 31, 2019, the first case of coronavirus disease 2019 (COVID-19), a severe infectious respiratory disease brought on by a new coronavirus (SARS-CoV-2), was reported in Wuhan, China (1). On March 11, 2020, the World Health Organization (WHO) formally designated it a global pandemic (2). Person-to-person transmission is *via* droplets released through coughing, sneezing, speaking, and contact with contaminated surfaces (3). In addition to raising concerns about global public health, COVID-19 also caused extraordinary disruptions to daily lives, with strong recommendations for social withdrawal, self-isolation, work interruptions, educational disruption, and travel limitations (4–7).

The COVID-19 pandemic has had detrimental psychological effects on healthcare workers who are on the front lines of treating infected patients (8). Personal and professional factors have contributed to fear, burnout, anxiety, depression, mental exhaustion, and insomnia among this population (4, 7). Some of the personal factors include a sense of fear, anxiety, and uncertainty, and a desire for appreciation, respect, and support, while work-related factors include unfamiliar responsibilities, insufficient resources, a lack of knowledge with personal protective equipment (PPE), and increased workloads (9).

Among the frontline healthcare workers are the pharmacists, who have been devotedly delivering essential services throughout the pandemic (10), and this has resulted in having them increasingly acknowledged as vital service providers around the world (11). This recognition stems from their ability to demonstrate that they are a dynamic workforce dedicated to public health and who can competently deliver a variety of services and products to meet current societal needs (12). During the lockdown, pharmacists were the most accessible healthcare members with whom patients could interact (13–16). They have actively participated in medical activities related to COVID-19 through utilizing their pharmacological expertise in screening, medication dispensing, as well as closely collaborating with other healthcare workers and governmental organizations to discover solutions and break down barriers (17, 18). Besides, they have played an essential role in community settings by raising public awareness related to health issues (19). As such, pharmacists are definitely frontline healthcare workers who are particularly positioned to provide care to a substantial segment of the population and have a high potential to contribute to the pandemic response.

While lockdown and staying at home regulations lingered, pharmacists continued working despite the numerous challenges. Hence, alongside this serious infectious public health event, the mental burden of pharmacists may be exacerbated by the rising number of confirmed and suspected cases, heavy workloads, negative emotions, exhaustion from PPE use, lack of certain medications, fear of spreading the disease to family, friends, and coworkers, as well as feelings of inadequate support (20, 21).

Due to the challenging conditions brought on by COVID-19, it has become crucial to evaluate the mental health of pharmacists who have been exposed to a variety of stress-related factors that have increased their stress levels, anxiety, depressive symptoms, and exacerbations of pre-existing mental disease (7, 20, 22, 23). In Lebanon, the COVID-19 pandemic, along with a severe economic downturn, has severely impacted an already struggling profession (24). The determination of the psychological impact of the COVID-19 regarding anxiety, insomnia, depression, and response to trauma on pharmacists in Lebanon during COVID-19, and the identification of characteristics that contribute to psychological distress, are warranted (25). Consequently, this study aimed to assess the psychological impact of COVID-19 regarding anxiety, insomnia, depression, and response to trauma on pharmacists in Lebanon during COVID-19, and to identify factors contributing to psychological distress.

## Methods

### Study design, setting, and participants

This was a cross-sectional study that involved pharmacists from all over Lebanon. Data were collected *via* an anonymous online questionnaire using Google Forms, over a period of 2 months during the COVID-19 outbreak from May 8 to July 5, 2020. A snowball sampling technique was used to collect data across the eight governorates (Mohafazat) of Lebanon and to target only Lebanese pharmacists who work in community, hospital, drug company, academic, and other settings.

### Sample size calculation

The minimal sample size was calculated using CDC’s Epi-Info for population surveys. The population size was set 13,000, which is the number of pharmacists in Lebanon. The expected frequency was set at 75%, which is the previously reported frequency of community pharmacists who are familiar with the recommendations of the Lebanese Order of Pharmacists (26). Consequently, a minimum sample of 282 pharmacists was required to produce a 95% confidence level and an acceptable margin of error of 5%.

### Data collection

The online questionnaire was developed in English language and piloted with 10 pharmacists to check for content and clarity. Amendments were then done and the final questionnaire was distributed forwarding its link to all pharmacists working in Lebanon. The study scope and purpose were explained at the beginning of the questionnaire, confirming that the survey was strictly confidential and conducted in compliance with the relevant data protection law. Participants were informed that their participation in the study is voluntary and they were assured that their responses would remain anonymous and confidential. They were requested to indicate through a mandatory selection box before beginning the survey that this was their first time doing so (ensuring 100% consent rate and preventing duplicate replies), and completion of the questionnaire until the end was considered as informed consent to participate.

### Ethical approval

The Ethics and Research Committee of the School of Pharmacy at the Lebanese International approved the study protocol (protocol number: 2020RC-042-LIUSOP) and followed the Declaration of Helsinki Ethical Principles for Medical Research Involving Human Subjects. The procedures pertaining to the anonymity of the data and the information provided to the volunteers were anticipated and the nature of the elements to be collected did not carry the risk of disclosing weaknesses unknown to the volunteer and thereby causing unpredictable reactions.

### Measurement tools

The questionnaire consisted of three sections. The first and second sections were concerned with the participants’ sociodemographic data and the pharmacists’ knowledge and concerns about COVID-19. In the following section, four validated scales that serve the purpose of the study in measuring the mental health status of the participants during the COVID-19 pandemic have been included. The 7-item Generalized Anxiety Disorder (GAD-7), 7-item Insomnia Severity Index (ISI), Patient Health Questionnaire 9-item depression module (PHQ-9), and Impact of Event Scale revised (IES-R) have been used to assess the psychological impact. The questionnaires were administered in English language because it is understood by the vast majority of Lebanese pharmacists who speak English. In fact, the OPL has adopted English as the language for their continuing education.

### The 7-item insomnia severity index

The 7-ISI is a self-reporting instrument composed of 7 items that was used to assess the nature, severity, and impact of insomnia. Each item is rated on a 5-point Likert scale from 0 to 4 with a total score ranging from 0 to 28. The total score was calculated and interpreted as follows: normal with no insomnia (0–7), subthreshold (8–14), moderate (15–21), and severe (22–28) insomnia (27, 28). The Cronbach’s alpha among our sample was 0.886.

### The patient health questionnaire 9-item depression module

Participants’ depressive symptoms during the COVID-19 pandemic were assessed using PHQ-9, which scores each of the nine Diagnostic and Statistical Manual of Mental Disorders, 4^th^ edition (DSM-IV) criteria of depression on a scale ranging from “0″ (not at all) to “3″ (nearly every day). The total sum of the responses ranging from 0 to 27 suggests varying levels of depression: no/minimal depression (0–4), mild (5–9), moderate (10–14), moderately severe (15–19), and severe (20–27) (29). The Cronbach’s alpha among our sample was 0.868.

### The 7-item generalized anxiety disorder scale

This 7-item self-rated tool was used to measure participants’ anxiety symptoms over the previous 2 weeks. Each item is assigned a score from “0″ (not at all) to “3″ (nearly every day). The total score of GAD-7 ranges from 0 to 21 and is grouped into four categories as follows: no/minimal anxiety (0–4), mild (5–9), moderate (10–14), and severe (15–21) (30). The Cronbach’s alpha among our sample was 0.930.

### Impact of event scale revised

The IES-R has been utilized as a self-report measure to assess the level of symptomatic response to specific traumatic events as the COVID-19 pandemic as it was manifested in the previous 7 days. It consisted of a brief self-administered 22-item questionnaire, and for response, a five-point Likert scale was used. Scale scoring of IES-R included a total score (ranging from 0 to 88) that indicated the global subjective stress regarding COVID-19. Higher levels of distress are reflected by higher total (or subscale) scores. The total IES-R score was divided into: absence of PTSD in those with little or no symptoms (0–23), being at risk with several symptoms (24–32), and probable PTSD (≥33) (31). The Cronbach’s alpha among our sample was 0.960.

### Statistical analysis

Descriptive statistics were performed to represent the participants’ characteristics, COVID-19 related information, the GAD-7 anxiety score, ISI insomnia severity score, PHQ-9 depression severity score, and IES-R global subjective stress score, and were expressed as percentages.

The GAD-7 anxiety score was expressed as “moderate and severe” versus “minimum and mild”; the insomnia severity index as “clinical insomnia (moderate and severe)” versus “no clinically significant insomnia and subthreshold insomnia”; the depression score as “moderate, moderately severe, and severe depression” versus “minimal and mild depression”; and the IES-R score as “little or no symptoms” versus “several symptoms” and “probable PTSD.” The bivariate associations between the scores and the participants’ characteristics were assessed using the Chi-square test for all variables or Fisher exact test (when >20% of the expected cell count were less than 5). Thereafter, four binary logistic regression models using the forward method were performed to preclude potential confounding. The dependent variables were the anxiety, insomnia, depression, and IES-R scores in the first, second, third, and fourth models, respectively. The participants’ characteristics having a *p*-value of less than 0.05 in the bivariate analysis were included as independent variables to account for them as potential confounding. The models were tested for adequacy in all the analysis. An alpha of 0.05 was used to determine statistical significance. All analyses were performed using the IBM’s Statistical Package for the Social Sciences (SPSS) version 22.0 (IBM, Inc., Chicago, IL).

## Results

### Demographic data of Lebanese pharmacists

A total of 311 Lebanese pharmacists participated in the study whose sociodemographic characteristics are shown in Table 1. The analyzed sample included participants from all Lebanese districts, of whom 251 (80.7%) were females and 181 (58.2%) aged between 26 and 35 years. As for the professional field, 57.2% work in community pharmacy, 6.1% work as hospital/clinical pharmacists, 14.8% in drug companies, 6.8% work in academia, 1% work in regulatory departments of pharmaceutical companies, and 14.1% in other pharmaceutical fields. Educational variables showed that 56.3% had a BPHARM degree and 43.7% had a BPHARM and postgraduate degrees (MS/PharmD/PhD). The majority (*n* = 112, 36%) belonged to the middle socioeconomic class with an average family income of more than 4,000,000 LBP per month.

**Table 1:** Participants’ Characteristics (*n* = 311).

		*n* (%)
Gender	Female	251 (80.7)
Age	18 to 25	77 (24.8)
	26 to 35	181 (58.2)
	36 to 45	34 (10.9)
	46 and above	19 (6.1)
Nationality	Lebanese	301 (96.8)
	Non-Lebanese	10 (3.2)
Area of residence	Beirut	72 (23.2)
	Baalbak-Hermel and Bekaa	98 (31.5)
	Mount Lebanon	74 (23.8)
	Nabatieh and South	49 (15.8)
	North and Akkar	18 (5.8)
Marital status	Single	179 (57.6)
	Married	128 (41.2)
	Widow/Divorced	4 (1.3)
Being employed	Yes	245 (78.8)
Field of work	Community pharmacist	178 (57.2)
	Hospital-based pharmacist	19 (6.1)
	Drug company	46 (14.8)
	Academia	21 (6.8)
	Regulatory	3 (1)
	Others	44 (14.1)
Degree	BPHARM	175 (56.3)
	BPHARM & (MS/PharmD/PhD)	136 (43.7)
Smoking cigarettes	Yes	23 (7.4)
Smoking more during COVID time	Yes	15 (65.2)
Smoking arguileh	Yes	85 (27.3)
Smoking more during COVID time	Yes	37 (43.5)
Consuming caffeinated beverage	Yes	271 (87.1)
Consuming more during COVID time	Yes	103 (38)
Consuming energy drinks	Yes	30 (9.6)
Consuming more during COVID time	Yes	10 (33.3)
Drinking alcohol	Yes	51 (16.4)
Drinking more during COVID time	Yes	10 (20)
Physical activity during lockdown	No	153 (49.2)
	2–3 times/week	117 (37.6)
	> 3 times per week	41 (13.2)
Drinking water during lockdown (≥ 2 Liters/day)	Yes	169 (54.3)
Family income per month in LBP	≤ 750,000	7 (2.3)
	[750,001 – 2,000,000]	95 (30.5)
	[2,000,001 – 4,000,000]	97 (31.2)
	> 4,000,000	112 (36)

COVID-19, Coronavirus disease 2019; LBP, Lebanese pounds; BPHARM, Bachelor of Pharmacy; MS, Master of Science; PharmD, Doctor of Pharmacy; PhD, Doctor of Philosophy.

### Lifestyle changes during COVID-19 time

More than half of the participants stated that they drank at least 2 liters of water per day and were physically active during quarantine. However, the lifestyle of Lebanese pharmacists was affected during the pandemic, where alcohol intake was increased by 20% among alcohol consumers (51 [16.4%]). As for smoking, 23 (7.4%) were cigarette while 85 (27.3%) were waterpipe smokers respectively, and more than half of those reported increased smoking during the COVID-19 time. Caffeinated beverages were consumed by 271 (87.1%) and less than half (38%) increased their consumption during COVID-19. Energy drinks consumption was reported by 30 (9.6%), of whom 33.3% also had raised consumption during the pandemic, while alcohol consumption was reported by 51 (16.4%) and almost 20% increased their consumption during COVID-19.

### Health and concerns about COVID-19, and results of the four mental health scales

Around half of the Lebanese pharmacists (*n* = 157, 50.5%) reported that they were in direct contact with infected/suspected COVID-19 patients during the pandemic period and 85 (27.3%) reported being frontline workers. Only 40 (12.9%) reported a history of chronic disease. The majority (*n* = 270, 86.8%) reported getting sufficient protection against COVID-19 infection during work and 196 (63%) reported that they were concerned about contracting COVID-19. A considerable proportion of participants had symptoms of anxiety (*n* = 128, 41.2%), insomnia (*n* = 64, 20.6%), depression (*n* = 157, 50.5%), and subjective stress (*n* = 227, 78.8%). Further details are shown in Table 2.

**Table 2:** Health and COVID-19 related information (*n* = 311).

		*n* (%)
Having any medical condition	Yes	40 (12.9)
Being diagnosed with COVID-19	Yes	0
Family members/colleagues diagnosed with COVID-19	Yes	15 (4.8)
Work involve contact with infected/suspected COVID-19 patients	Yes	157 (50.5)
Directly engaged in clinical activities with patients having elevated temperature or confirmed COVID-19	No – Second-line worker	226 (72.7)
	Yes – Frontline worker	85 (27.3)
Getting sufficient protection against COVID-19 infection during work	Yes	270 (86.8)
Hours reading COVID-19 updates (past week)	<1 h/day	169 (54.3)
	1–2 h/day	110 (35.4)
	3–4 h/day	24 (7.7)
	5 or more hours per day	8 (2.6)
Getting sufficient support (state, community, and social media) during COVID-19	No Yes Somehow	112 (36.5) 78 (25.4) 117 (38.1)
Worried about getting COVID-19 infection	Yes	196 (63)
GAD-7 anxiety score	No / Minimal	82 (26.4)
	Mild	101 (32.5)
	Moderate	72 (23.2)
	Severe	56 (18)
ISI insomnia score	No clinically significant insomnia	138 (44.4)
	Subthreshold insomnia	109 (35)
	Clinical insomnia (moderate)	54 (17.4)
	Clinical insomnia (severe)	10 (3.2)
Sleeping hours / night	<5	43 (13.8)
	5 to 7	188 (60.5)
	8 to 10	76 (24.4)
	>10	4 (1.3)
Taking naps during the day	Yes	76 (24.4)
PHQ-9 depression severity score	No / Minimal depression	54 (17.4)
	Mild depression	100 (32.2)
	Moderate depression	97 (31.2)
	Moderately severe	39 (12.5)
	Severe depression	21 (6.8)
IES-R	Little or no symptoms	61 (21.2)
	Several symptoms	131 (45.5)
	Probable PTSD	96 (33.3)

COVID-19, Coronavirus disease 2019; GAD, 7-item Generalized Anxiety Disorder; ISI, 7-item Insomnia Severity Index; PHQ-9, Patient Health Questionnaire 9-item depression module; IES-R, Impact of Events Scale-Revised; PTSD, Post-traumatic stress disorder.

### Scores of measurement and association of possible influence factors with anxiety, insomnia, depression, and stress during COVID-19 pandemic

Numerous factors were shown to be significantly associated to anxiety, insomnia, depression, and stress among the pharmacists in Lebanon in the bivariate analysis. Tables 3–6 show the association of possible influence factors with GAD, insomnia, depression, and stress during COVID-19 pandemic.

**Table 3:** Bivariate and multivariable associations for GAD-7 anxiety scale.

		Bivariate analysis		Multivariable analysis *n* = 311	
		Minimum & Mild (*n* = 183) *n* (%)	Moderate & Severe (*n* = 128) *n* (%)	aOR (95% CI)	*p*-value
Gender	Male	39 (65)	21 (35)	-	
	Female	144 (57.4)	107 (42.6)		
Age	18 to 25	37 (48.1)	40 (51.9)	-	
	26 to 35	112 (61.9)	69 (38.1)		
	36 to 45	24 (70.6)	10 (29.4)		
	46 and above	10 (52.6)	9 (47.4)		
Nationality	Lebanese	174 (57.8)	127 (42.2)*	Reference	0.2
	Non-Lebanese	9 (90)	1 (10)	0.25 (0.03; 2.07)	
Area of residence	Beirut	40 (55.6)	32 (44.4)	-	
	Baalbak-Hermel and Bekaa	62 (63.3)	36 (36.7)		
	Mount Lebanon	35 (47.3)	39 (52.7)		
	Nabatieh and South	32 (65.3)	17 (34.7)		
	North and Akkar	14 (77.8)	4 (22.2)		
Marital status	Single	1q2	84 (46.9)*	Reference	
	Married	86 (67.2)	42 (32.8)	0.51 (0.31; 0.83)	0.007
	Widow/Divorced	2 (50)	2 (50)	0.98 (0.13; 7.29)	0.9
Being employed	No	37 (56.1)	29 (43.9)	-	
	Yes	146 (59.6)	99 (40.4)		
Field of work	Community pharmacist	105 (59)	73 (41)	-	
	Hospital-based pharmacist	11 (57.9)	8 (42.1)		
	Drug company	24 (52.2)	22 (47.8)		
	Academia	15 (71.4)	6 (28.6)		
	Regulatory	1 (33.3)	2 (66.7)		
	Others	27 (61.4)	17 (38.6)		
Degree	BPHARM	106 (60.6)	69 (39.4)	-	
	BPHARM & (MS/PharmD/PhD)	77 (56.6)	59 (43.4)		
Smoking cigarettes	No	172 (59.7)	116 (40.3)	-	
	Yes	11 (47.8)	12 (52.2)		
Smoking arguileh	No	129 (57.1)	97 (42.9)	-	
	Yes	54 (63.5)	31 (36.5)		
Consuming caffeinated beverage	No	23 (57.5)	17 (42.5)	-	
	Yes	160 (59)	111 (41)		
Consuming energy drinks	No	166 (59.1)	115 (40.9)	-	
	Yes	17 (56.7)	13 (43.3)		
Drinking alcohol	No	157 (60.4)	103 (39.6)	-	
	Yes	26 (51)	25 (49)		
Physical activity during lockdown	No	90 (58.8)	63 (41.2)	-	
	2–3 times/week	69 (59)	48 (41)		
	>3 times per week	24 (58.5)	17 (41.5)		
Drinking water during lockdown (≥ 2 Liters/day)	No	79 (55.6)	63 (44.4)	-	
	Yes	104 (61.5)	65 (38.5)		
Family income per month in LBP	≤750,000	3 (42.9)	4 (57.1)	-	
	[750,001 – 2,000,000]	51 (53.7)	44 (46.3)		
	[2,000,001 – 4,000,000]	60 (61.9)	37 (38.1)		
	>4,000,000	69 (61.6)	43 (38.4)		
Having any medical condition	No	161 (59.4)	110 (40.6)	-	
	Yes	22 (55)	18 (45)		
Family members/colleagues diagnosed with COVID-19	No	171 (57.8)	125 (42.2)	-	
	Yes	12 (80)	3 (20)		
Work involve contact with infected/suspected COVID-19 patients	No Yes	101 (65.6) 82 (52.2)	53 (34.4) * 75 (47.8)	Reference 1.73 (1.08; 2.78)	0.02
Directly engaged in clinical activities with patients having elevated temperature or confirmed COVID-19	No	137 (60.6)	89 (39.4)	-	
		46 (54.1)	39 (45.9)		
	Yes				
Getting sufficient protection against COVID-19 infection during work	No	19 (46.3)	22 (53.7)	-	
	Yes	164 (60.7)	106 (39.3)		
Hours reading COVID-19 updates (past week)	<1 h/day	107 (63.3)	62 (36.7)	-	
	1–2 h / day 3–4 h /day 5 or more hours per day	61 (55.5) 11 (45.8) 4 (50)	49 (44.5) 13 (54.2) 4 (50)		
Getting sufficient support (state, community, and social media) during COVID-19	No	64 (57.1)	48 (42.9)	-	
	Yes Somehow	48 (61.5) 69 (59)	30 (38.5) 48 (41)		
Worried about getting COVID-19 infection	No	82 (71.3)	33 (28.7)**	Reference	0.001
	Yes	101 (51.5)	95 (48.5)	2.35 (1.40; 3.94)	

Logistic regression model assumptions met: Omnibus test likelihood ratio chi-square *p* < 0.001; Hosmer-Lemeshow test for goodness of fit *p* > 0.05. COVID-19, Coronavirus disease 2019; GAD, 7-item Generalized Anxiety Disorder; aOR, Adjusted Odds ratio; CI, Confidence Interval; BPHARM, Bachelor of Pharmacy; MS, Master of Science; PharmD, Doctor of Pharmacy; PhD, Doctor of Philosophy.

* *p*  < 0.05 (bivariate analysis).

** *p*  < 0.01 (bivariate analysis).

**Table 4:** Bivariate and multivariable associations for Insomnia severity score (ISI).

		Bivariate analysis		Multivariable analysis *n* = 311	
		No clinical/subthreshold *n* = 247 *n* (%)	Moderate/Severe insomnia n = 64 *n* (%)	aOR (95% CI)	*p*-value
Gender	Male	51 (85)	9 (15)	-	
	Female	196 (78.1)	55 (21.9)		
Age	18 to 25	55 (71.4)	22 (28.6)	-	
	26 to 35	148 (81.8)	33 (18.2)		
	36 to 45	28 (82.4)	6 (17.6)		
	46 and above	16 (84.2)	3 (15.8)		
Nationality	Lebanese	237 (78.7)	64 (21.3)	-	
	Non-Lebanese	10 (100)	0		
Area of residence	Beirut	59 (81.9)	13 (18.1)	-	
	Baalbak-Hermel and Bekaa	82 (83.7)	16 (16.3)		
	Mount Lebanon	52 (70.3)	22 (29.7)		
	Nabatieh and South	38 (77.6)	11 (22.4)		
	North and Akkar	16 (88.9)	2 (11.1)		
Marital status	Single	136 (76)	43 (24)	-	
	Married	107 (83.6)	21 (16.4)		
	Widow/Divorced	4 (100)	0		
Being employed	No	45 (68.2)	21 (31.8)*	Reference	0.006
	Yes	202 (82.4)	43 (17.6)	0.42 (0.22; 0.78)	
Field of work	Community pharmacist	148 (83.1)	30 (16.9)	-	
	Hospital-based pharmacist	14 (73.7)	5 (26.3)		
	Drug company	33 (71.7)	13 (28.3)		
	Academia	18 (85.7)	3 (14.3)		
	Regulatory	2 (66.7)	1 (33.3)		
	Others	32 (72.7)	12 (27.3)		
Degree	BPHARM	145 (82.9)	30 (17.1)	-	
	BPHARM & (MS/PharmD/PhD)	102 (75)	34 (25)		
Smoking cigarettes	No	230 (79.9)	58 (20.1)	-	
	Yes	17 (73.9)	6 (26.1)		
Smoking arguileh	No	182 (80.5)	44 (19.5)	-	
	Yes	65 (76.5)	20 (23.5)		
Consuming caffeinated beverage	No	34 (85)	6 (15)	-	
	Yes	213 (78.6)	58 (21.4)		
Consuming energy drinks	No	224 (79.7)	57 (20.3)	-	
	Yes	23 (76.7)	7 (23.3)		
Drinking alcohol	No	203 (78.1)	57 (21.9)	-	
	Yes	44 (86.3)	7 (13.7)		
Physical activity during lockdown	No	121 (79.1)	32 (20.9)	-	
	2–3 times/week	95 (81.2)	22 (18.8)		
	>3 times per week	31 (75.6)	10 (24.4)		
Drinking water during lockdown (≥ 2 Liters/day)	No	106 (74.6)	36 (25.4)	-	
	Yes	141 (83.4)	28 (16.6)		
Family income per month in LBP	≤750,000	5 (71.4)	2 (28.6)	-	
	[750,001–2,000,000]	76 (80)	19 (20)		
	[2,000,001–4,000,000]	72 (74.2)	25 (25.8)		
	>4,000,000	94 (83.9)	18 (16.1)		
Having any medical condition	No	222 (81.9)	49 (18.1)**	Reference	0.003
	Yes	25 (62.5)	15 (37.5)	2.99 (1.45; 6.19)	
Family members/colleagues diagnosed with COVID-19	No	232 (78.4)	64 (21.6)		
	Yes	15 (100)	0		
Work involve contact with infected/suspected COVID-19 patients	No Yes	120 (77.9) 127 (80.9)	34 (22.1) 30 (19.1)		
Directly engaged in clinical activities with patients having elevated temperature or confirmed COVID-19	No	181 (80.1)	45 (19.9)		
				
	Yes	66 (77.6)	19 (22.4)		
Getting sufficient protection against COVID-19 infection during work	No	31 (75.6)	10 (24.4)		
	Yes	216 (80)	54 (20)		
Hours reading COVID-19 updates (past week)	<1 h/day	133 (78.7)	36 (21.3)		
	1–2 h/day 3–4 h/day 5 or more hours per day	90 (81.8) 18 (75) 6 (75)	20 (18.2) 6 (25) 2 (25)		
Getting sufficient support (state, community, and social media) during COVID-19	No	87 (77.7)	25 (22.3)		
	Yes Somehow	64 (82.1) 94 (80.3)	14 (17.9) 23 (19.7)		
Worried about getting COVID-19 infection	No	98 (85.2)	17 (14.8)		
	Yes	149 (76)	47 (24)		

Logistic regression model assumptions met: Omnibus test likelihood ratio chi-square *p* < 0.001; Hosmer-Lemeshow test for goodness of fit *p* > 0.05. COVID-19, Coronavirus disease 2019; ISI, 7-item Insomnia Severity Index; aOR, Adjusted Odds ratio; CI, Confidence Interval; BPHARM, Bachelor of Pharmacy; MS, Master of Science; PharmD, Doctor of Pharmacy; PhD, Doctor of Philosophy.

* *p* < 0.05 (bivariate analysis).

** *p* < 0.01 (bivariate analysis).

**Table 5:** Bivariate and multivariable associations for PHQ-9 depression severity score.

		Bivariate analysis		Multivariable analysis *n* = 290	
		Minimum & Mild *n* = 154 *n* (%)	Moderate/moderately Severe & Severe *n* = 136 *n* (%)	aOR (95% CI)	*p*-value
Gender	Male	28 (48.3)	30 (51.7)	-	
	Female	126 (54.3)	106 (45.7)		
Age	18 to 25	30 (42.9)	40 (57.1)	-	
	26 to 35	95 (56.2)	74 (43.8)		
	36 to 45	18 (56.3)	14 (43.8)		
	46 and above	11 (57.9)	8 (42.1)		
Nationality	Lebanese	146 (52)	135 (48)*	Reference	0.049
	Non-Lebanese	8 (88.9)	1 (11.1)	0.07 (0.005; 0.99)	
Area of residence	Beirut	34 (51.5)	32 (48.5)	-	
	Baalbak-Hermel and Bekaa	49 (52.7)	44 (47.3)		
	Mount Lebanon	33 (50.8)	32 (49.2)		
	Nabatieh and South	24 (50)	24 (50)		
	North and Akkar	14 (77.8)	4 (22.2)		
Marital status	Single	79 (47.3)	88 (52.7)*	Reference	
	Married	71 (59.7)	48 (40.3)	0.69 (0.40; 1.19)	0.2
	Widow/Divorced	4 (100)	0	0	
Being employed	No	25 (41)	36 (59)*	Reference	0.003
	Yes	129 (56.3)	100 (43.7)	0.35 (0.18; 0.69)	
Field of work	Community pharmacist	90 (53.9)	77 (46.1)	-	
	Hospital-based pharmacist	7 (36.8)	12 (63.2)		
	Drug company	23 (54.8)	19 (45.2)		
	Academia	12 (57.1)	9 (42.9)		
	Regulatory	1 (50)	1 (50)		
	Others	21 (53.8)	18 (46.2)		
Degree	BPHARM	86 (51.8)	80 (48.2)	-	
	BPHARM & (MS/PharmD/PhD)	68 (54.8)	56 (45.2)		
Smoking cigarettes	No	146 (54.5)	122 (45.5)	-	
	Yes	8 (36.4)	14 (63.6)		
Smoking arguileh	No	117 (54.9)	96 (45.1)	-	
	Yes	37 (48.1)	40 (51.9)		
Consuming caffeinated beverage	No	19 (48.7)	20 (51.3)	-	
	Yes	135 (53.8)	116 (46.2)		
Consuming energy drinks	No	146 (55.5)	117 (44.5)*	Reference	0.002
	Yes	8 (29.6)	19 (70.4)	5.37 (1.81; 15.9)	
Drinking alcohol	No	130 (53.9)	111 (46.1)	-	
	Yes	24 (49)	25 (51)		
Physical activity during lockdown	No	66 (47.5)	73 (52.5)	-	
	2–3 times/week	66 (59.5)	45 (40.5)		
	> 3 times per week	22 (55)	18 (45)		
Drinking water during lockdown (≥ 2 Liters/day)	No	59 (44.7)	73 (55.3)**	Reference	0.02
	Yes	95 (60.1)	63 (39.9)	0.52 (0.31; 0.88)	
Family income per month in LBP	≤ 750,000	3 (42.9)	4 (57.1)	-	
	[750,001 – 2,000,000]	46 (51.1)	44 (48.9)		
	[2,000,001 – 4,000,000]	49 (56.3)	38 (43.7)		
	> 4,000,000	56 (52.8)	50 (47.2)		
Having any medical condition	No	140 (55.6)	112 (44.4)*	Reference	0.1
	Yes	14 (36.8)	24 (63.2)	2.00 (0.87; 4.61)	
Family members/colleagues diagnosed with COVID-19	No	142 (51.6)	133 (48.4)*	Reference	0.03
	Yes	12 (80)	3 (20)	0.17 (0.03; 0.80)	
Work involve contact with infected/suspected COVID-19 patients	No Yes	87 (60) 67 (46.2)	58 (40) * 78 (53.8)	Reference 3.06 (1.73; 5.39)	<0.001
Directly engaged in clinical activities with patients having elevated temperature or confirmed COVID-19	No	115 (53.7)	99 (46.3)	-	
				
	Yes	39 (51.3)	37 (48.7)		
Getting sufficient protection against COVID-19 infection during work	No	13 (33.3)	26 (66.7)**	Reference	0.003
	Yes	141 (56.2)	110 (43.8)	0.28 (0.12; 0.64)	
Hours reading COVID-19 updates (past week)	<1 h/day	83 (52.9)	74 (47.1)	-	
	1–2 h / day 3–4 h /day 5 or more hours per day	55 (53.4) 13 (59.1) 3 (37.5)	48 (46.6) 9 (40.9) 5 (62.5)		
Getting sufficient support (state, community, and social media) during COVID-19	No	55 (53.4)	48 (46.6)	-	
	Yes Somehow	38 (52.1) 59 (53.6)	35 (47.9) 51 (46.4)		
Worried about getting COVID-19 infection	No	73 (65.2)	39 (34.8)**	Reference	0.001
	Yes	81 (45.5)	97 (54.5)	2.64 (1.49; 4.67)	

Logistic regression model assumptions met: Omnibus test likelihood ratio chi-square *p* < 0.001; Hosmer-Lemeshow test for goodness of fit *p* > 0.05. COVID-19, Coronavirus disease 2019; PHQ-9, Patient Health Questionnaire 9-item depression module; aOR, Adjusted Odds ratio; CI, Confidence Interval; BPHARM, Bachelor of Pharmacy; MS, Master of Science; PharmD, Doctor of Pharmacy; PhD, Doctor of Philosophy.

* *p*  < 0.05 (bivariate analysis).

** *p*  < 0.01 (bivariate analysis).

**Table 6:** Bivariate and multivariable associations for IES-R.

		Bivariate analysis		Multivariable analysis *n* = 288	
		Little or several symptoms *n* = 192 *n* (%)	Probable PTSD *n* = 96 *n* (%)	aOR (95% CI)	*p*-value
Gender	Male	34 (65.4)	18 (34.6)	-	
	Female	158 (66.9)	78 (33.1)		
Age	18 to 25	44 (60.3)	29 (39.7)	-	
	26 to 35	111 (66.1)	57 (33.9)		
	36 to 45	22 (73.3)	8 (26.7)		
	46 and above	15 (88.2)	2 (11.8)		
Nationality	Lebanese	186 (66.2)	95 (33.8)	-	
	Non-Lebanese	6 (85.7)	1 (14.3)		
Area of residence	Beirut	40 (60.6)	26 (39.4)	-	
	Baalbak-Hermel and Bekaa	64 (68.8)	29 (31.2)		
	Mount Lebanon	39 (59.1)	27 (40.9)		
	Nabatieh and South	35 (76.1)	11 (23.9)		
	North and Akkar	14 (82.4)	3 (17.6)		
Marital status	Single	102 (61.1)	65 (38.9)	-	
	Married	87 (74.4)	30 (25.6)		
	Widow/Divorced	3 (75)	1 (25)		
Being employed	No	36 (59)	25 (41)	-	
	Yes	156 (68.7)	71 (31.3)		
Field of work	Community pharmacist	111 (66.1)	57 (33.9)	-	
	Hospital-based pharmacist	13 (76.5)	4 (23.5)		
	Drug company	27 (61.4)	17 (38.6)		
	Academia	13 (81.3)	3 (18.8)		
	Regulatory	2 (66.7)	1 (33.3)		
	Others	26 (65)	14 (35)		
Degree	BPHARM	113 (67.3)	55 (32.7)	-	
	BPHARM & (MS/PharmD/PhD)	79 (65.8)	41 (34.2)		
Smoking cigarettes	No	180 (67.4)	87 (32.6)	-	
	Yes	12 (57.1)	9 (42.9)		
Smoking arguileh	No	146 (69.2)	65 (30.8)	-	
	Yes	46 (59.7)	31 (40.3)		
Consuming caffeinated beverage	No	22 (61.1)	14 (38.9)	-	
	Yes	170 (67.5)	82 (32.5)		
Consuming energy drinks	No	176 (68)	83 (32)	-	
	Yes	16 (55.2)	13 (44.8)		
Drinking alcohol	No	166 (68.6)	76 (31.4)	-	
	Yes	26 (56.5)	20 (43.5)		
Physical activity during lockdown	No	94 (65.3)	50 (34.7)	-	
	2–3 times/week	74 (69.8)	32 (30.2)		
	> 3 times per week	24 (63.2)	14 (36.8)		
Drinking water during lockdown (≥2 Liters/day)	No	95 (69.3)	42 (30.7)	-	
	Yes	97 (64.2)	54 (35.8)		
Family income per month in LBP	≤ 750,000	3 (42.9)	4 (57.1)	-	
	[750,001 – 2,000,000]	63 (67.7)	30 (32.3)		
	[2,000,001 – 4,000,000]	63 (70.8)	26 (29.2)		
	> 4,000,000	63 (63.6)	36 (36.4)		
Having any medical condition	No	169 (67.6)	81 (32.4)	-	
	Yes	23 (60.5)	15 (39.5)		
Family members/colleagues diagnosed with COVID-19	No	184 (66.4)	93 (33.6)	-	
	Yes	8 (72.7)	3 (27.3)		
Work involve contact with infected/suspected COVID-19 patients	No Yes	108 (74.5) 84 (58.7)	37 (25.5) ** 59 (41.3)	Reference 1.86 (1.11; 3.14)	0.02
Directly engaged in clinical activities with patients having elevated temperature or confirmed COVID-19	No	150 (70.1)	64 (29.9)*	Reference	0.2
				
	Yes	42 (56.8)	32 (43.2)	1.47 (0.83; 2.60)	
Getting sufficient protection against COVID-19 infection during work	No	23 (57.5)	17 (42.5)	-	
	Yes	169 (68.1)	79 (31.9)		
Hours reading COVID-19 updates (past week)	<1 h/day	114 (69.9)	49 (30.1)	-	
	1–2 h/day 3–4 h/day 5 or more hours per day	63 (64.9) 11 (55) 4 (50)	34 (35.1) 9 (45) 4 (50)		
Getting sufficient support (state, community, and social media) during COVID-19	No	68 (64.8)	37 (35.2)	-	
	Yes Somehow	47 (67.1) 73 (67)	23 (32.9) 36 (33)		
Worried about getting COVID-19 infection	No	72 (72.7)	27 (27.3)	-	
	Yes	120 (63.5)	69 (36.5)		

Logistic regression model assumptions met: Omnibus test likelihood ratio chi-square *p* < 0.001; Hosmer-Lemeshow test for goodness of fit *p* > 0.05. COVID-19, Coronavirus disease 2019; IES-R, Impact of Events Scale-Revised; PTSD, Post-traumatic stress disorder; aOR, Adjusted Odds ratio; CI, Confidence Interval; BPHARM, Bachelor of Pharmacy; MS, Master of Science; PharmD, Doctor of Pharmacy; PhD, Doctor of Philosophy.

* *p*  < 0.05 (bivariate analysis).

** *p*  < 0.01 (bivariate analysis).

### Anxiety

Being married was significantly associated with lower anxiety levels as compared to single status (aOR: 0.51, 95% CI: 0.31; 0.83, *p*-value: 0.007). Interestingly, higher anxiety scores were significantly associated with pharmacists who expressed concern about contracting COVID-19 infection (aOR: 2.35, 95% CI: 1.40; 3.94, p-value: 0.001) and those who reported that their work involves contact with infected/suspected COVID-19 patients (aOR: 1.73, 95% CI: 1.08; 2.78, *p*-value: 0.02) respectively.

### Insomnia

Higher rates of insomnia were associated with the presence of chronic medical disorders. Having a medical condition was significantly associated with greater scores for insomnia compared to those who did not have co-morbidities (aOR: 2.99, 95% CI: 1.45; 6.19, *p*-value: 0.003). On the other hand, being employed was significantly associated with lower levels of insomnia (aOR: 0.42, 95% CI: 0.22; 0.78, *p*-value: 0.006).

### Depression

Interestingly, higher depression scores were significantly associated with pharmacists who expressed concern about contracting COVID-19 infection (aOR: 2.64, 95% CI: 1.49; 4.67, *p*-value: 0.001) and those who reported that their work involves contact with infected/suspected COVID-19 patients (aOR: 3.06, 95% CI: 1.73; 5.39, *p*-value: 0.001). Regarding variations to lifestyle, consuming energy drinks (aOR: 5.37, 95% CI: 1.81; 15.9, *p*-value: 0.002) was found to be significantly associated with higher depression scores. However, drinking more than 2 liters of water per day during lockdown (aOR: 0.52, 95% CI: 0.31; 0.88, *p*-value: 0.02) and receiving appropriate COVID-19 infection prevention at work (aOR: 0.28, 95% CI: 0.12; 0.64, p-value: 0.003) were protective factors towards depression. Similarly, being employed was significantly associated with lower depression scores (aOR: 0.35, 95% CI: 0.18; 0.69, *p*-value: 0.003).

### Stress

Notably, pharmacists who stated that their work involves contact with COVID-19 patients who are infected or suspected of being infected, were substantially more likely to report greater stress levels (aOR: 1.86, 95% CI: 1.11; 3.14, *p*-value: 0.02).

## Discussion

This study is among the very few ones addressing mental health in Lebanon during COVID-19 pandemic, and is the first to explore the effect of the pandemic on mental health of pharmacists. Most of the sociodemographic characteristics of the analyzed sample were aligned with previously published reports about pharmacists in Lebanon (32). Even though there aren’t many studies on this subject in this particular population, it was deemed suitable to compare and discuss the findings based on research on healthcare workers as well as general population surveys.

High levels of stress, insomnia, anxiety, and depression were observed among Lebanese pharmacists. A number of variables including poor pharmacists’ wages, which are typically not commensurate with their skills, as well as internal political upheaval and high inflation, could be to blame (33, 34). In fact, on November 19, 2020, Lebanon was ranked as the second-highest nation in terms of global inflation (Lebanon: 365%), only after Venezuela (2,133%). The pandemic, which exacerbated the burden on pharmacists’ mental health, made this situation much worse. This may eventually continue to reduce pharmacists’ productivity, performance, and optimism (34). The anxiety, insomnia, depression, and stress findings in this population are also in conformity with reported high prevalence among healthcare workers according to results of a systematic review and meta-analysis published in 2021 (35).

Our findings showed that throughout the pandemic, there was an increase in the respondents’ unhealthy behaviors. The rates of alcohol consumption, smoking, and caffeinated and energy drinks were increased and this was earlier reported among the Lebanese population during the pandemic (36), probably as a strategy to react to stressful situations, and has been reported elsewhere (37, 38). The increase in smoking and drinking alcohol during stressful experiences is actually consistent with the “coping effect” of tobacco versus psychological stress (39, 40).

Lower anxiety was noted among married individuals, which is consistent with a previous study by Lawal and colleagues, which reported better coping among married females (41). However, Tan and Colleagues reported that single people experience less of the negative effects of events, stress, anxiety, and depression than divorced, separated, or widowed individuals (42). On the other hand, those with chronic medical diseases were shown to experience high levels of mental health distress. This was in line with the findings of other studies that have documented COVID-19 fatalities in patients with comorbidities (43).

The current study is in line with an extensive body of evidence which indicated that consumption of energy drinks was increased (36, 44) and significantly associated with depression symptoms (45). Notable preventive factors against depression included appropriate COVID-19 protection at work and good hydration. To maintain better immune systems and reduce the risk of chronic illnesses and infectious diseases, drinking water is essential (46). Lack of access to PPE has been directly linked to psychological distress and depressive symptoms (47), although having enough PPE might lessen the potential negative effects of COVID exposure on mental health (48). Healthcare workers would feel safer with PPE as this relates to their own health, that of their patients, and that of their loved ones (48). Similarly, lower insomnia and depression scores were noted among employed pharmacists. This appears logical in a country with collapsed economy and the need for relative financial security through having a job.

As previously documented in a recent study among Portuguese pharmacists, increased anxiety and stress scores were reported with pharmacists who claimed that their profession entails contact with infected/suspected COVID-19 patients (49). Furthermore, anxiety and depression scores were much higher in pharmacists who showed fear about developing COVID-19 infection, possibly highlighting the severe and wide-ranging psychological effects that the pandemic can have on daily life (50). The increase in the number of patients seen, the amount of triage done, the bulk of COVID-19 information delivered, the vast medication shortages, and the amount of workplace harassment, may have all affected the mental health of Lebanese pharmacists in the studied sample.

### Limitations

This study, which was based on an electronic survey, has several limitations. First of all, it is difficult to determine the temporal link between exposure and outcomes from the cross-sectional design of the study. Second, the snowball sampling technique may have been associated with a possible risk of selection bias, which may affect the generalizability of the current findings. However, it is believed that this risk is minimized as our sample included pharmacists from districts all over Lebanon, which can provide some generalizability to the current findings. Moreover, although a web-based survey is one of the most efficient ways to obtain quick data, especially if social interactions are prohibited (i.e., lockdown), it may be associated with another possible risk of selection bias as it may have excluded senior pharmacists with lesser digital literacy. Further research is still recommended to include a broader range of pharmacists and evaluate their mental health amid pandemics as COVID-19 and influenzae to confirm the current findings. However, recall bias may still arise as a consequence of self-reporting assessment methods. Nevertheless, it is believed that this bias is minimized as the survey utilized validated scales with good internal consistency and using the English language that is well understood by all Lebanese pharmacists. Finally, the current study applied generic validated scales rather than COVID-19 specific scales to assess mental health. Further studies are recommended to minimize the current biases.

### Practical implications and future directions

The preliminary results from pharmacists in Lebanon reflect increase in stress, burden, and frustration felt by pharmacists, creating a negative impact on their mental health and well-being in the wake of the global pandemic. As frontline healthcare workers, the role of pharmacists in the community should not be overlooked, and their mental health should be well investigated. As we move towards a post-pandemic world, pertinent information about the mental health of pharmacists and associated factors should come up with essential recommendations on how to assist pharmacists in coping with anxiety, insomnia, depression, and stress occurring as collateral effects of COVID-19. With pharmacists being an integral segment of the healthcare system, their mental health and well-being is vital for proper health services. Accordingly, the impact of this study shall relate to pharmacists’ community role, and should help to purposefully interpret their ability to effectively serve their community.

## Conclusion

During the COVID-19 pandemic, pharmacists played a crucial role in providing treatment on the front lines, but the increasing demand for their services also led to changes in their mental health. Throughout the pandemic, pharmacists have remained essential to the efficient provision of healthcare. The findings indicated that the COVID-19 pandemic and the ensuing lockdown had a significant negative influence on the mental health of Lebanese pharmacists. Therefore, it is as important for pharmacy professionals to put their own mental health first as it is for them to assist their patients. They must take the time to assess their own mental health for symptoms of burnout and general psychological strain in order to continue providing the best care for patients. It is important to keep in mind that stress is a typical response to a global infectious disease pandemic, however, it is crucial to prepare for stress and take the necessary actions to reduce its negative effects on the body and mind. A well-equipped workforce may be maintained by efforts towards a regular schedule, engaging in fun activities, asking for assistance from coworkers and family, and creating stress-reduction techniques. To make sure that enough resources and assistance are being offered, pharmacy managers should maintain frequent and regular communication with their staff. In light of the ongoing challenges brought on by COVID-19, it is imperative to treat the mental health of healthcare workers. The development of guidelines and solutions to enhance the safety of pharmacists and other healthcare workers during future pandemics and upcoming global outbreaks should be a top priority for pharmacy organizations.

## Data availability statement

The original contributions presented in the study are included in the article/supplementary material, further inquiries can be directed to the corresponding author.

## Ethics statement

The Ethics and Research Committee of the School of Pharmacy at the Lebanese International approved the study protocol (protocol number: 2020RC-042- LIUSOP) and followed the Declaration of Helsinki Ethical Principles for Medical Research Involving Human Subjects. The participants provided their written informed consent to participate in this study.

## Author contributions

JS and DH: study conception and design. FSa, EB, and FSaa: acquisition of data. ZA, JS, MA, and FSaa: analysis and interpretation of results. MD, SY, and NM: investigation. MC, DH, and ZA: methodology. JS, SY, and DH: project administration. SY and JS: resources. ZA: software. DH and MA: visualization. JS, DH, and SY: writing original manuscript draft. SY, MR, DH, FSa, and MA: revision and editing it critically for important intellectual content. FSa and DH: validation. MR: supervision. All authors contributed to the article and approved the submitted version.

## Conflict of interest

The authors declare that the research was conducted in the absence of any commercial or financial relationships that could be construed as a potential conflict of interest.

## Publisher’s note

All claims expressed in this article are solely those of the authors and do not necessarily represent those of their affiliated organizations, or those of the publisher, the editors and the reviewers. Any product that may be evaluated in this article, or claim that may be made by its manufacturer, is not guaranteed or endorsed by the publisher.
